# Student ultrasound education, current view and controversies. Role of Artificial Intelligence, Virtual Reality and telemedicine

**DOI:** 10.1186/s13089-024-00382-5

**Published:** 2024-09-27

**Authors:** Nils Daum, Michael Blaivas, Adrian Goudie, Beatrice Hoffmann, Christian Jenssen, Ricarda Neubauer, Florian Recker, Tudor Voicu Moga, Constantinos Zervides, Christoph Frank Dietrich

**Affiliations:** 1 Department of Anesthesiology and Intensive Care Medicine (CCM/CVK), Charité – Universitätsmedizin Berlin, corporate member of Freie Universität Berlin and Humboldt Universität Zu Berlin, Berlin, Germany; 2 Brandenburg Institute for Clinical Ultrasound (BICUS) at Brandenburg Medical University, Neuruppin, Germany; 3 Department of Medicine, University of South Carolina School of Medicine, Columbia, SC USA; 4 Fiona Stanley Hospital, Perth, Australia; 5 Department of Emergency Medicine, Beth Israel Deaconess Medical Center, Harvard Medical School, Boston, MA USA; 6 Department for Internal Medicine, Krankenhaus Märkisch Oderland, Strausberg, Germany; 7 University Hospital Bonn, Bonn, Germany; 8 Department of Obstetrics and Prenatal Medicine, University Hospital Bonn, Bonn, Germany; 9 Department of Gastroenterology and Hepatology, “Victor Babeș” University of Medicine and Pharmacy, Piața Eftimie Murgu 2, 300041 Timișoara, Romania; 10 Center of Advanced Research in Gastroenterology and Hepatology, “Victor Babeș” University of Medicine and Pharmacy, 300041 Timisoara, Romania; 11 CZMH Limassol Medical Physics and Dosimetry Services LTD, Limassol, Cyprus; 12 Department Allgemeine Innere Medizin (DAIM), Kliniken Hirslanden Beau Site, Salem und Permanence, Bern, Switzerland

**Keywords:** Artificial Intelligence, Education, Students, Telemedicine, Ultrasound

## Abstract

The digitization of medicine will play an increasingly significant role in future years. In particular, telemedicine, Virtual Reality (VR) and innovative Artificial Intelligence (AI) systems offer tremendous potential in imaging diagnostics and are expected to shape ultrasound diagnostics and teaching significantly. However, it is crucial to consider the advantages and disadvantages of employing these new technologies and how best to teach and manage their use. This paper provides an overview of telemedicine, VR and AI in student ultrasound education, presenting current perspectives and controversies.

## Introduction

Incorporating ultrasound education into medical schools has become widespread worldwide [1–3]. This is reflected in a rapidly growing body of literature [4–11]. Both the European Federation of Societies for Ultrasound in Medicine and Biology (EFSUMB) [2] and the World Federation of Ultrasound in Medicine and Biology (WFUMB) issued position papers stating: “*that ultrasound should be used systematically as an easily accessible and instructive educational tool in the curriculum of modern medical schools*” [1, 12]. Furthermore, the Society of Ultrasound Medical Education (SUSME) and the World Interactive Network Focused on Critical Ultrasound (WINFOCUS) recently published an international consensus statement regarding this topic [11].

This article series aims to develop a core ultrasound curriculum. Such a core curriculum should be a step-by-step open-access living document that is steadily improving and expanding. The clinical topics and pathologies taught each semester should be reflected in the ultrasound course content. Arriving at a consensus on a core curriculum will be difficult because of national differences in regulations, responsibilities, and curricula for medical studies and many different medical specialties, experiences, and opinions. This paper series is structured according to the categories of controversies in student ultrasound education (SUSE) and provides a discussion platform to allow input from universities, teachers, students, and regulatory bodies to debate these issues. The first part introduces the subject and provides a brief history of SUSE. Other parts discuss the role of teachers, students (including peer student teaching) and sonographers, didactic structures, knowledge and skill evaluation (theoretical and practical examination of learning success) and certification, learning materials, and surroundings, including skills labs and simulation, the role of measurements and knowledge of reference values, teaching pathological findings during SUSE, ethical issues and misinterpretations, and teaching interventional procedures.

## Role of Artificial Intelligence in student ultrasound education

In medical imaging, Artificial Intelligence (AI) represents a paradigm shift, particularly with the advent of deep learning algorithms capable of creating models directly from raw data. Ultrasound, as a widely accessible imaging modality, benefits significantly from AI integration, particularly with adjunct sonographic imaging features such as elastography, Doppler, and contrast applications.

AI is a term widely used to describe machine capabilities that simulate human intelligence [13]. It have gained traction across various industries, including medicine, driven by advancements in processing power, notably the development of graphics processing units (GPUs) [14]. Enhancing the algorithms' performance involves training the models with additional data, which can refine their analysis processes. However, in scenarios where the algorithm impacts actual patient care, known as "software as a medical device," such enhancements occur through offline training rather than real-time adjustments.

Despite the benefits, the utilization of AI in medicine poses limitations and risks, as demonstrated by Lee et al. through their study involving chatbots like ChatGPT to address medical inquiries [15]. In the realm of medical imaging, a notable "paradigm shift" is underway due to significant advancements in AI. Deep learning algorithms, characterized by their intricate networks of artificial neurons, represent the pinnacle of data-driven models. They possess the unique ability to construct models directly from raw data, rendering them especially adept for image analysis [16]. Unlike human interpretation, AI algorithms possess the capability to discern patterns that may elude imaging specialists.

Ultrasound stands apart from other imaging techniques because it is the most widely available cross-sectional imaging worldwide and is likely the most cost-effective. In advanced ultrasound systems, AI software is already assuming a pivotal role in image analysis by offering crucial applications [17]. These applications encompass pathology detection, diagnosis or classification, guidance, and segmentation, thereby bolstering the accuracy of image analysis and supporting physicians in their diagnostic endeavor. For instance, an AI tool highlighting malignancy in focal liver lesions evaluated by contrast-enhanced ultrasound can improve the diagnostic performance of a novice, reaching the results of an expert sonographer [18]. Similarly, a computer-aided-diagnosis (CAD) for thyroid and breast lesions have been shown to augment diagnostic performance, elevating the accuracy of inexperienced clinicians to that of seasoned radiologists [19–21].

Due to ultrasound's reliance on the operator's skill and spatial abilities, AI algorithms can offer support to inexperienced operators by advising on probe handling and the identification of organs, vessels and other landmark structures. The AI's information on whether an organ has been thoroughly examined can further assist the examination process [22–27].

New technologies have been integrated into radiology workflow in clinical practice and education, in part driven by pressures for increased efficiency and cost-effectiveness [28, 29]. Considering the rapid growth of AI applications in medical imaging, some warnings were sounded regarding the possible impact of AI on radiology as a profession, estimating a progressive replacement of radiologists or at least a significant restructuring of the work processes [30–32]. Nevertheless, it is widely agreed that radiologists should continue to receive training, including education on new AI technologies [29, 33]. Any transitions or adjustments to new AI-driven imaging paradigms will likely occur over many years. In a recent publication, Wang et al. reviewed medical students’ attitudes toward AI. In their study, they pointed out that a minority of students thought AI would replace the radiologist, but a majority feared AI would decrease the standards of their profession. Survey data shows that the anticipated impact of AI has deterred medical students from pursuing radiology as a career and has caused anxiety among those who had considered it a future profession [29]. In the same review, they revealed that students who received AI-based teaching were more prone to pursue a radiology profession than those without AI education. Nevertheless, most medical students agree that AI training would enhance their careers and think that AI training should be part of their university education [34, 35]. Recently, the available literature on medical students' expectations about the impact of AI on medical imaging, training in diagnostic imaging, and the associated changes in the professional profile of radiologists and ultrasound specialists has been comprehensively summarized [29, 36].

AI can be utilized in medical imaging and by extrapolation in ultrasound to address various issues, such as decreasing missed diagnoses, increasing diagnostic precision, enhancing reconstructed image quality, and uncovering image features that cannot be seen with the eye.

### Arguments supporting AI in student education

- More time for teaching and research. By having AI take over repetitive tasks, physicians` workloads can be decompressed, with the resulting time savings being available for teaching medical students or residents and focusing on research [17].
- AI software can handle vast amounts of input data, thus providing ample training opportunities. Experienced practitioners may accumulate vast knowledge throughout their careers by interpreting images. However, the number of cases on which they can base their decisions is limited. In comparison, computers can be trained on massive datasets and have impeccable recall [28]. AI may assist in proper probe positioning and valid measurements in learning ultrasound techniques [23, 24]. Especially for ultrasound procedures that are difficult to learn, it has already been shown that using AI can facilitate trainees' recognition of sonographic anatomy and shorten their learning curve [25–27].
- AI supports student-tailored ultrasound education. AI has numerous potential applications in ultrasound education, including the ability to gather large amounts of data on medical student and resident education, performance, and progress, customize education based on individual learning styles and needs, and enable precise, targeted education in ultrasound [37]. Moreover, the various aspects of educational AI, such as chatting features and real-time discussion of topics, along with AI algorithms that can guide students through an entire examination, depict anatomy, and test pathology knowledge in the process, can all combine to replace many in-person teaching requirements.
- Creates opportunities to combine teaching with different interactive games. Using AI in ultrasound education offers new ways to engage medical students and enhance their learning efficiency. The gamification of ultrasound training is becoming more prevalent in radiology education programs [38].
- Evaluate, follow-up, and offer feedback to students during the image acquisition. AI can first analyze a case and assign it to a student/resident who has reached certain milestones in their learning trajectory. Later, it can assist students in examining related case reports and relevant literature. After discussion and review, the case will be added to the teaching file, and the competency profile can be updated as part of a real-time teaching file catalog [37, 39].
- Reducing inequity by reducing barriers and minimizing operator and patient dependency. AI-based ultrasound systems can help reduce barriers and provide a convenient way to access ultrasound education and services in many regions/countries through cloud systems (DICOM data bank), where educational medical resources are imbalanced. These cutting-edge technologies have significantly improved our understanding of ultrasonic image variations caused by factors such as operator, scanner, and patient dependence [40].

### Arguments against AI in medical student education

- With AI’s growth, physicians’ knowledge and skills to perform specific tasks would likely decline. Relying increasingly on AI, medical students may not develop the knowledge and understanding that accumulates by repeating basic tasks. There is a question of whether a physician should rely as much on AI-powered software for these tasks without first understanding and performing them independently [41]. If AI-based training is not regulated correctly during training, it can lead to excessive reliance on AI, thus weakening the trainee’s ability to perform and interpret ultrasound. It could pose potential challenges if AI tools are introduced to learners prematurely, lacking adequate evaluation, and devoid of human supervision and scrutiny to assess their advantages or limitations. In that case, inexperienced physicians may become overly reliant on AI tools, believing they are "superior" to manual effort and human cognitive work [42].
- Lack of control of AI in ultrasound novices. Inexperienced users cannot identify AI errors. Another challenge in educating future physicians is instructing trainees on identifying AI errors and recognizing the more elusive and unpredictable AI mistakes [43, 44].
- “Garbage in, garbage out” theory. Human input is minimal in AI-based software, apart from annotating each image or case in the training data with the correct diagnosis. This approach eliminates the need for pre-identifying and extracting image features. However, data quality can significantly impact AI algorithm training efficiency and performance. Unfortunately, patient records, medical registries, and ultrasound images are not always compiled into accurate and complete datasets, which in some cases might generate biases or decrease performance potential [14].
- Fear of being replaced. Research has shown that the anticipated impact of AI has deterred medical students from pursuing radiology as a career and has even caused anxiety among those who still wish to choose it as their future path [29, 34, 45]. It is vital to educate students that any workforce changes are likely to occur slowly and will come with additional knowledge that will be required from physicians.
- Lack of empathy in AI-based education. The absence of empathy in AI-driven education may foster a disconnect between students and their learning experiences, potentially diminishing motivation and engagement. The automation of educational processes can exacerbate this disconnect, hindering students' development of emotional intelligence and interpersonal skills crucial for personal and professional growth.

### Discussion

The field of ultrasound permits a wide range of AI-based applications. AI can help reduce repetitive tasks, increase the supervisor’s available time, and provide supervision in the future. This allows educators to focus on tasks of higher value, such as spatial aptitude, learning habits, research, and communication skills [29, 46, 47].

Along with interpreting ultrasound images, trainees must also acquire the ability to understand the processes of AI-generated results, potential pitfalls and errors. If physicians want to remain masters of diagnostic procedures supported by AI in the future, students must be allowed to acquire fundamental competency in data science and AI. Therefore, the rapid integration of AI as learning content in medical school curricula must be prioritized [48].

In the meantime, students and residents should become familiar with AI algorithms and understand the reasons, situations, and circumstances where AI tools may not perform as intended. By embracing AI, we can improve our performance, enhance patient care and medical education.

## Role of Virtual Reality in student ultrasound education

### Introduction

There has been a recent dramatic change in the way how medical education can be delivered. Internet and mobile devices offer a vast amount of information and learning possibilities. In the era of AI, immersive technologies are evolving providing a whole new universe of teaching opportunities. These technologies comprising Augmented Reality (AR) and Virtual Reality (VR) already transform medical education and how it is delivered to future health care professionals [49]. The use of VR in health professions education has increased dramatically in recent years. VR simulation training has been mandatory in different educational fields such as aviation for many decades. In medical education simulation training gained more importance and a more mandatory status in the last two decades. A stress-free learning environment can be created by VR, in which learners can repeatedly train standard physiological situations as well as pathological “patient”-cases.

In classical VR, the user puts on a head-mounted display (HMD) to join a simulated environment, with virtual characters. In immersive VR, the user puts on the HMD, is situated in a virtual environment, and additionally uses either haptic controllers or hand-tracking devices to interact with the environment. The immersive setup provides a content-rich experience that can improve user engagement and thus, the training outcome. The application of immersive VR ultrasound teaching has been successfully applied in point of care ultrasound (POCUS) teaching settings [50, 51].

Some systems use one single haptic device, e.g. ultrasound probe in combination with VR glasses, some systems do use the same in combination with phantoms. Haptic devices often allow measuring forces applied. VR is used to train specific ultrasound-guided skills such as regional anesthesia or fine needle puncture [52]. The latter requires cognitive-motor skills for the performance of regional anesthesia under ultrasound guidance.

Due to greater achievements in graphic technology and AI more and more detailed 3-and 4-dimensional images are generated for VR ultrasound training, such as echocardiography [53]. The latter will certainly lead to a growing use of immersive VR in medical ultrasound education with a good acceptance among students offered VR education [54]. Reviews and meta-analysis found a significant effect on VR simulation in skill acquisition and knowledge gain in medical education, yet research regarding the effects of VR simulation related to ultrasound training are still sparse though.

### Arguments supporting VR Ultrasound education

- Repetitive training possibilities can be provided with immersive VR ultrasound teaching. When VR glasses and probes are available in a higher amount, students might even be able to lend them and train at home anytime and anywhere.
- Adaptive simulation settings with increasing competence levels can be created in immersive VR ultrasound education settings. Different modes enable the learning independently to adapt to progressive learning task.
- Safe learning atmosphere is established when learners do have the ability to independently learn on their own solely or together with other students.
- Possible remote supervision might be a good option to give instant feedback with the training sessions and due to the VR—based scenario student and supervisor can meet within immersive VR anytime, anywhere.

### Arguments against VR Ultrasound education

- Lacking supervision is one possible limitation when it comes to VR training as students might often be left alone in their further training. A structured course- based training in combination with VR-educational sessions upfront to any independent training sessions might limit failures and frustration due to lacking input to a minimum.
- High costs of VR glasses and software are a major limitation to give a high number of students access to VR training at the same time.
- Cognitive overload by immersive VR and how it influences sustained medical learning and retention of motor performance are still part of current research [55].
- Real life haptic and personal interaction are missing and main limiting factors when it comes to any kind of virtual or simulation-based training in medical education. Personal interaction within training sessions is important to improve soft skills and personal development.

### Discussion

VR can be described as a form of high-fidelity simulation that may be used to enhance the quality of medical education. Scientific studies exploring the effects of immersive VR ultrasound education are increasing but still sparse. With the HMD being portable and applicable everywhere, VR training enables learners to train and test their abilities literally independent of time and space. Once scenarios are completed, students receive virtual debriefing and feedback on their performance.

A major limitation of VR training is the fact that it causes increased cognitive load, especially when learning complex skills. VR training has been shown to increase cognitive load decrease retention in motor memory when compared to classical screen- based learning approaches. The same study also showed that cognitive load decreased over time, indicating that users may have to adapt to immersive VR training [55]. It could also be shown that repeated and distributed VR training causes a lower cognitive load and should therefore be induced when the learning situation is increased in complexity [56].

Overall VR ultrasound education is an effective method to be integrated into existing ultrasound curricula to enhance and expand training possibilities yet to be distributed reasonably to avoid cognitive overload with its possible negative correlation on motor learning effects and retention.

## Role of telemedicine in student ultrasound education

Practical clinical skills are essential components of everyday medical practice. In recent years, this realization has led to redesigning theoretically oriented standard courses to emphasize practical orientation. For ultrasound training, practical implementation is an essential part of being able to produce and interpret ultrasound images. Due to the SARS-CoV-2 pandemic and the resulting contact restrictions, the training of this practical content became severely restricted, causing a sudden shift to digital teaching [57–59].

Teledidactic applications in ultrasound education can take many forms, including real-time videoconferencing, store-and-forward imaging, and web-based educational resources. Real-time videoconferencing is one of ultrasound education's most promising virtual applications [60]. Teledidactic approaches can connect students in remote or underserved regions with experts in urban areas [61].

Web-based resources are another valuable approach to ultrasound education [62]. Online courses and interactive simulations provide a flexible and accessible learning environment for students. Such courses can be accessed from anywhere with an internet connection and are designed to meet student's specific learning objectives. Interactive simulations provide a safe and realistic environment for students to practice ultrasound techniques and procedures, allowing repeated practice without requiring live patients.

Several studies investigated approaches to teaching practical ultrasound skills via an online platform and demonstrated the effectiveness of virtual ultrasound education [63–65]. Using e-learning videos showed a learning advantage over text-based e-learning units [66]. Sometimes, online simulations or videos showed better results than traditional learning courses [67–69]. A combination of learning courses supplemented with instructional videos also showed a positive effect [70]. When comparing online simulations with pure video lessons, knowledge acquired through a simulation lasts longer [71]. However, many of these approaches were based on blended-learning concept, and students criticized the lack of interaction opportunities [72–74]. Some evaluations showed that students are open to online teaching opportunities and prefer additional online material over exclusive face-to-face teaching [73].

Ultrasound education via exclusive online training has some limitations, especially concerning practical learning experience, a crucial component of diagnostic ultrasound. Nevertheless, it is possible to convey theoretical and practical content to students via specific online formats or interactive synchronous online tutorials [75]. Incorporating new techniques, such as different camera perspectives, can benefit online learning formats [39].

### Arguments supporting teledidactic approaches in student ultrasound education

- Fast and easy access, regardless of location. The primary teaching method for recognizing structures and pathologies involves visual diagnoses using ultrasound images, which can be taught via online courses. Additionally, knowledge assessment can be conducted through online resources, enabling interactive testing and reinforcing understanding.
- New teaching formats. Recent advancements in synchronous online teaching formats have expanded their capacity to effectively impart theoretical concepts and practical skills in a digital format. This enables comprehensive learning experiences that bridge the gap between theoretical understanding and hands-on application [75].
- New online camera perspectives**.** Emerging online formats can display the patient's transducer position and sonographic settings. This feature enhances the learning experience by visually understanding how ultrasounds are performed and interpreted in a clinical setting.
- Cost efficiency. Costs for many devices can be saved through a higher number of participants in an online tutorial.

### Arguments against teledidactic approaches in student ultrasound education

- Missing hands-on experience**.** Ultrasound training inherently relies on hands-on experience in acquiring and interpreting ultrasound images. This hands-on engagement is indispensable for learners to develop proficiency and confidence in using ultrasound technology effectively in clinical settings. For some students, telemedicine education may have to include remote hands-on teaching/monitoring for learners who do not have local supervisors for hands-on learning.
- New, but challenging teaching methods. Teaching online formats demands a nuanced blend of technical expertise and specialized pedagogical knowledge. Successfully conveying hands-on skills virtually necessitates a deep understanding of both the technical aspects of the subject matter and effective instructional methodologies. Adapting traditional teaching methods into engaging online formats is imperative while ensuring learners gain practical proficiency despite the digital learning environment.
- Missing patient interaction. Teledidactic approaches usually fail to provide direct patient interaction and practical hands-on experience. While they excel in delivering theoretical knowledge, the absence of direct patient engagement limits the opportunity for learners to apply their skills in real-life scenarios. This gap in practical experience can challenge fully preparing individuals for the intricacies and nuances involved in patient care and clinical practice.

### Discussion

The development of teledidactic approaches in ultrasound education represents a forward-thinking method to bolster and augment the teaching of ultrasound. Techniques such as real-time video conferencing, the use of store-and-forward imaging, and the availability of web-based learning resources offer substantial educational benefits to students. As telemedicine continues to expand and gain traction, innovative and effective methods for ultrasound training are expected to proliferate. By tapping into the potential of telemedicine, we can guarantee access to superior ultrasound education for students everywhere, overcoming barriers related to their physical location or specific conditions. Teaching practical skills purely through online formats is subject to limitations. The independent performance of specific examination techniques on actual patients is essential to any practical learning content, especially in ultrasound training. However, because of the SARS-CoV-2 pandemic, innovations, such as tele-guided ultrasound courses, have been established in online teaching to impart practical knowledge to students [76].

Despite this progress, there is still a need for significant improvements in digital restructuring and training in digital media to comprehensively deliver such courses. However, fundamental ultrasound training skills can be effectively complemented with online formats, offering the opportunity to convey knowledge through interactive teaching methods.

### Summary

The incorporation of ultrasound education into medical schools is supported by a significant global trend and endorsements from major federations and societies [77]. This movement is bolstered by an increasing body of literature and consensus statements from organizations such as EFSUMB, WFUMB, and SUSME, emphasizing the importance of ultrasound in medical curricula. Advancement toward a core ultrasound curriculum offers a structured and systematic approach to teaching, enhancing the educational experience. Integrating advanced technologies such as AI in ultrasound education is a notable strength, offering efficient learning, tailored educational experiences, and the ability to handle extensive training data.

However, this integration also has weaknesses, notably the potential over-reliance on AI, which could lead to a decline in fundamental skills and knowledge among students. The variability in ultrasound interpretation and its operator-dependent nature also present limitations. Additionally, the diversity of opinions and experiences across disciplines and institutions might complicate reaching a consensus on a core curriculum, potentially hindering standardization.

On the opportunity front, incorporating ultrasound education opens doors for revolutionizing teaching methods with AI like VR, making them more interactive and engaging. It aligns with the growing trend toward practical, hands-on learning in medical education, providing a relevant skill set for students.

Conversely, one of the primary threats is the potential impact of AI on the role of educators and traditional teaching methods, which might lead to resistance or apprehension. The rapid advancement of technology also threatens to create a gap between current teaching methods and future needs, requiring continuous curriculum updates and adaptability. In addition, there are risks of misinterpretation and ethical concerns related to AI in medical education, necessitating careful management to ensure effective and appropriate technology use.

In summary, while the integration of ultrasound education into medical curricula is underpinned by solid support and presents significant opportunities for educational advancement, it also faces challenges and risks that need careful consideration to ensure successful and effective integration into medical education.

## Data Availability

Availability of data and materials is not required due to the study design.

## References

[1] Dietrich CF, Hoffmann B, Abramowicz J, Badea R, Braden B, Cantisani V et al (2019) Medical student ultrasound education: a WFUMB Position Paper. Part I Ultrasound Med Biol 45(2):271–281. 10.1016/j.ultrasmedbio.2018.09.017 30497768 10.1016/j.ultrasmedbio.2018.09.017

[2] Cantisani V, Dietrich CF, Badea R, Dudea S, Prosch H, Cerezo E et al (2016) EFSUMB statement on medical student education in ultrasound [long version]. Ultrasound Int Open 2(1):E2-7. 10.1055/s-0035-1569413 27689163 10.1055/s-0035-1569413 PMC5023223

[3] Dietrich CF, Sirli RL, Barth G, Blaivas M, Daum N, Dong Y et al (2024) Student ultrasound education - current views and controversies. Ultraschall Med. 10.1055/a-2265-1070 38484782 10.1055/a-2265-1070

[4] Davis JJ, Wessner CE, Potts J, Au AK, Pohl CA, Fields JM (2018) Ultrasonography in undergraduate medical education: a systematic review. J Ultrasound Med 37(11):2667–2679. 10.1002/jum.14628 29708268 10.1002/jum.14628

[5] Feilchenfeld Z, Dornan T, Whitehead C, Kuper A (2017) Ultrasound in undergraduate medical education: a systematic and critical review. Med Educ 51(4):366–378. 10.1111/medu.13211 28118684 10.1111/medu.13211

[6] Kenny EJG, Makwana HN, Thankachan M, Clunie L, Duenas AN (2022) The use of ultrasound in undergraduate medical anatomy education: a systematic review with narrative synthesis. Med Sci Educ 32(5):1195–1208. 10.1007/s40670-022-01593-y 36276779 10.1007/s40670-022-01593-y PMC9583998

[7] Duarte ML, Santos LRD, Iared W, Peccin MS (2022) Comparison of ultrasonography learning between distance teaching and traditional methodology. An educational systematic review. Sao Paulo Med J 140(6):806–817. 10.1590/1516-3180.2021.1047.R.19052022 36043680 10.1590/1516-3180.2021.1047.R.19052022 PMC9671565

[8] DeBiasio C, Pageau P, Shefrin A, Woo MY, Cheung WJ (2023) Point-of-Care-ultrasound in undergraduate medical education: a scoping review of assessment methods. Ultrasound J 15(1):30. 10.1186/s13089-023-00325-6 37302105 10.1186/s13089-023-00325-6 PMC10258183

[9] Tarique U, Tang B, Singh M, Kulasegaram KM, Ailon J (2018) Ultrasound curricula in undergraduate medical education: a scoping review. J Ultrasound Med 37(1):69–82. 10.1002/jum.14333 28748549 10.1002/jum.14333

[10] Birrane J, Misran H, Creaney M, Shorten G, Nix CMA (2018) scoping review of ultrasound teaching in undergraduate medical education. Med Sci Educ 28:45–56. 10.1007/s40670-017-0491-4

[11] Hoppmann RA, Mladenovic J, Melniker L, Badea R, Blaivas M, Montorfano M et al (2022) International consensus conference recommendations on ultrasound education for undergraduate medical students. Ultrasound J 14(1):31. 10.1186/s13089-022-00279-1 35895165 10.1186/s13089-022-00279-1 PMC9329507

[12] Hoffmann B, Blaivas M, Abramowicz J, Bachmann M, Badea R, Braden B et al (2020) Medical Student Ultrasound Education, a WFUMB Position Paper, Part II. A consensus statement of ultrasound societies. Med Ultrason 22(2):220–229. 10.11152/mu-2599 32399529 10.11152/mu-2599

[13] McCorduck P (2004) Machines who think, 2nd edn. A. K. Peters, Natick

[14] Cantisani V, Grani G, Tovoli F, Piscaglia F, Catalano C (2020) Artificial intelligence: what is it and how can it expand the ultrasound potential in the future? Ultraschall Med 41(4):356–360. 10.1055/a-1173-4315 32750719 10.1055/a-1173-4315

[15] Lee P, Bubeck S, Petro J (2023) Benefits, limits, and risks of GPT-4 as an AI chatbot for medicine. N Engl J Med 388(13):1233–1239. 10.1056/NEJMsr2214184 36988602 10.1056/NEJMsr2214184

[16] Hinton G (2018) Deep learning-a technology with the potential to transform health care. JAMA 320(11):1101–1102. 10.1001/jama.2018.11100 30178065 10.1001/jama.2018.11100

[17] Kim YH (2021) Artificial intelligence in medical ultrasonography: driving on an unpaved road. Ultrasonography 40(3):313–317. 10.14366/usg.21031 34053212 10.14366/usg.21031 PMC8217795

[18] Moga TV, Popescu A, Sporea I, Danila M, David C, Gui V et al (2017) Is contrast enhanced ultrasonography a useful tool in a beginner’s hand? How much can a computer assisted diagnosis prototype help in characterizing the malignancy of focal liver lesions? Med Ultrasonogr. 19(3):7. 10.11152/mu-936 10.11152/mu-936 28845489

[19] Li X, Zhang S, Zhang Q, Wei X, Pan Y, Zhao J et al (2019) Diagnosis of thyroid cancer using deep convolutional neural network models applied to sonographic images: a retrospective, multicohort, diagnostic study. Lancet Oncol 20(2):193–201. 10.1016/s1470-2045(18)30762-9 30583848 10.1016/S1470-2045(18)30762-9 PMC7083202

[20] Fresilli D, Grani G, De Pascali ML, Alagna G, Tassone E, Ramundo V et al (2020) Computer-aided diagnostic system for thyroid nodule sonographic evaluation outperforms the specificity of less experienced examiners. J Ultrasound 23(2):169–174. 10.1007/s40477-020-00453-y 32246401 10.1007/s40477-020-00453-y PMC7242558

[21] Di Segni M, de Soccio V, Cantisani V, Bonito G, Rubini A, Di Segni G et al (2018) Automated classification of focal breast lesions according to S-detect: validation and role as a clinical and teaching tool. J Ultrasound 21(2):105–118. 10.1007/s40477-018-0297-2 29681007 10.1007/s40477-018-0297-2 PMC5972107

[22] Biswas M, Kuppili V, Edla DR, Suri HS, Saba L, Marinhoe RT et al (2018) Symtosis: a liver ultrasound tissue characterization and risk stratification in optimized deep learning paradigm. Comput Methods Programs Biomed 155:165–177. 10.1016/j.cmpb.2017.12.016 29512496 10.1016/j.cmpb.2017.12.016

[23] Ferraz S, Coimbra M, Pedrosa J (2023) Assisted probe guidance in cardiac ultrasound: a review. Front Cardiovasc Med 10:1056055. 10.3389/fcvm.2023.1056055 36865885 10.3389/fcvm.2023.1056055 PMC9971589

[24] Schneider M, Bartko P, Geller W, Dannenberg V, König A, Binder C et al (2021) A machine learning algorithm supports ultrasound-naïve novices in the acquisition of diagnostic echocardiography loops and provides accurate estimation of LVEF. Int J Cardiovasc Imaging 37(2):577–586. 10.1007/s10554-020-02046-6 33029699 10.1007/s10554-020-02046-6 PMC7541096

[25] Yao L, Zhang C, Xu B, Yi S, Li J, Ding X et al (2023) A deep learning-based system for mediastinum station localization in linear EUS (with video). Endosc Ultrasound 12(5):417–423. 10.1097/eus.0000000000000011 37969169 10.1097/eus.0000000000000011 PMC10631614

[26] Yao L, Zhang J, Liu J, Zhu L, Ding X, Chen D et al (2021) A deep learning-based system for bile duct annotation and station recognition in linear endoscopic ultrasound. EBioMedicine 65:103238. 10.1016/j.ebiom.2021.103238 33639404 10.1016/j.ebiom.2021.103238 PMC7921468

[27] Zhang J, Zhu L, Yao L, Ding X, Chen D, Wu H et al (2020) Deep learning-based pancreas segmentation and station recognition system in EUS: development and validation of a useful training tool (with video). Gastrointest Endosc 92(4):874–85.e3. 10.1016/j.gie.2020.04.071 32387499 10.1016/j.gie.2020.04.071

[28] Gore JC (2020) Artificial intelligence in medical imaging. Magn Reson Imaging 68:A1-a4. 10.1016/j.mri.2019.12.006 31857130 10.1016/j.mri.2019.12.006

[29] Wang C, Xie H, Wang S, Yang S, Hu L (2023) Radiological education in the era of artificial intelligence: a review. Medicine 102(1):e32518. 10.1097/md.0000000000032518 36607870 10.1097/MD.0000000000032518 PMC9829296

[30] Mazurowski MA (2019) Artificial intelligence may cause a significant disruption to the radiology workforce. J Am Coll Radiol 16(8):1077–1082. 10.1016/j.jacr.2019.01.026 30975611 10.1016/j.jacr.2019.01.026

[31] Thrall JH, Li X, Li Q, Cruz C, Do S, Dreyer K et al (2018) Artificial intelligence and machine learning in radiology: opportunities, challenges, pitfalls, and criteria for success. J Am Coll Radiol 15(3 Pt B):504–508. 10.1016/j.jacr.2017.12.026 29402533 10.1016/j.jacr.2017.12.026

[32] Recht M, Bryan RN (2017) Artificial intelligence: threat or boon to radiologists? J Am Coll Radiol 14(11):1476–1480. 10.1016/j.jacr.2017.07.007 28826960 10.1016/j.jacr.2017.07.007

[33] Tajmir SH, Alkasab TK (2018) Toward augmented radiologists: changes in radiology education in the era of machine learning and artificial intelligence. Acad Radiol 25(6):747–750. 10.1016/j.acra.2018.03.007 29599010 10.1016/j.acra.2018.03.007

[34] Sit C, Srinivasan R, Amlani A, Muthuswamy K, Azam A, Monzon L et al (2020) Attitudes and perceptions of UK medical students towards artificial intelligence and radiology: a multicentre survey. Insights Imaging 11(1):14. 10.1186/s13244-019-0830-7 32025951 10.1186/s13244-019-0830-7 PMC7002761

[35] Leandros S, Pelagia K-K, Marina S, Constantinos Z (2021) Exploring medical students’ and faculty’s perception on artificial intelligence and robotics. A questionnaire survey. J Artif Intell Med Sci. 2(1–2):76–84. 10.2991/jaims.d.210617.002

[36] Santomartino SM, Yi PH (2022) Systematic review of radiologist and medical student attitudes on the role and impact of AI in radiology. Acad Radiol 29(11):1748–1756. 10.1016/j.acra.2021.12.032 35105524 10.1016/j.acra.2021.12.032

[37] Duong MT, Rauschecker AM, Rudie JD, Chen PH, Cook TS, Bryan RN et al (2019) Artificial intelligence for precision education in radiology. Br J Radiol 92(1103):20190389. 10.1259/bjr.20190389 31322909 10.1259/bjr.20190389 PMC6849670

[38] Awan O, Dey C, Salts H, Brian J, Fotos J, Royston E et al (2019) Making learning fun: gaming in radiology education. Acad Radiol 26(8):1127–1136. 10.1016/j.acra.2019.02.020 31005406 10.1016/j.acra.2019.02.020

[39] Boten DN, Daum N, Schutz T, Spethmann S (2023) From videogames to teaching — different camera perspectives in an interactive synchronous online tutorial. Med Sci Educ. 10.1007/s40670-023-01833-9 37886296 10.1007/s40670-023-01833-9 PMC10597901

[40] Shen YT, Chen L, Yue WW, Xu HX (2021) Artificial intelligence in ultrasound. Eur J Radiol 139:109717. 10.1016/j.ejrad.2021.109717 33962110 10.1016/j.ejrad.2021.109717

[41] Nguyen GK, Shetty AS (2018) Artificial intelligence and machine learning: opportunities for radiologists in training. J Am Coll Radiol 15(9):1320–1321. 10.1016/j.jacr.2018.05.024 29941242 10.1016/j.jacr.2018.05.024

[42] Challen R, Denny J, Pitt M, Gompels L, Edwards T, Tsaneva-Atanasova K (2019) Artificial intelligence, bias and clinical safety. BMJ Qual Saf 28(3):231–237. 10.1136/bmjqs-2018-008370 30636200 10.1136/bmjqs-2018-008370 PMC6560460

[43] Rodriguez-Ruiz A, Lång K, Gubern-Merida A, Teuwen J, Broeders M, Gennaro G et al (2019) Can we reduce the workload of mammographic screening by automatic identification of normal exams with artificial intelligence? A feasibility study. Eur Radiol 29(9):4825–4832. 10.1007/s00330-019-06186-9 30993432 10.1007/s00330-019-06186-9 PMC6682851

[44] Ho CWL, Soon D, Caals K, Kapur J (2019) Governance of automated image analysis and artificial intelligence analytics in healthcare. Clin Radiol 74(5):329–337. 10.1016/j.crad.2019.02.005 30898383 10.1016/j.crad.2019.02.005

[45] van Hoek J, Huber A, Leichtle A, Härmä K, Hilt D, von Tengg-Kobligk H et al (2019) A survey on the future of radiology among radiologists, medical students and surgeons: students and surgeons tend to be more skeptical about artificial intelligence and radiologists may fear that other disciplines take over. Eur J Radiol 121:108742. 10.1016/j.ejrad.2019.108742 31734640 10.1016/j.ejrad.2019.108742

[46] Masters K (2019) Artificial intelligence in medical education. Med Teach 41(9):976–980. 10.1080/0142159x.2019.1595557 31007106 10.1080/0142159X.2019.1595557

[47] Chan KS, Zary N (2019) Applications and challenges of implementing artificial intelligence in medical education: integrative review. JMIR Med Educ 5(1):e13930. 10.2196/13930 31199295 10.2196/13930 PMC6598417

[48] Seth P, Hueppchen N, Miller SD, Rudzicz F, Ding J, Parakh K et al (2023) Data science as a core competency in undergraduate medical education in the age of artificial intelligence in health care. JMIR Med Educ 9:e46344. 10.2196/46344 37432728 10.2196/46344 PMC10369309

[49] Mergen M, Meyerheim M, Graf N (2023) Reviewing the current state of virtual reality integration in medical education – a scoping review protocol. Syst Rev. 10.1186/s13643-023-02266-6 37337293 10.1186/s13643-023-02266-6 PMC10280972

[50] Andersen NL, Jensen RO, Konge L, Laursen CB, Falster C, Jacobsen N et al (2023) Immersive virtual reality in basic point-of-care ultrasound training: a randomized controlled trial. Ultrasound Med Biol 49(1):178–185. 10.1016/j.ultrasmedbio.2022.08.012 36216656 10.1016/j.ultrasmedbio.2022.08.012

[51] Junge K, Larsen JD, Stougaard SW, Jensen RO, Falster C, Posth S et al (2024) Education in focused assessment with sonography for trauma using immersive virtual reality: a prospective, interventional cohort study and non-inferiority analysis with a historical control. Ultrasound Med Biol 50(2):277–284. 10.1016/j.ultrasmedbio.2023.10.013 38040522 10.1016/j.ultrasmedbio.2023.10.013

[52] Chuan A, Qian J, Bogdanovych A, Kumar A, Mckendrick M, Mcleod G (2023) Design and validation of a virtual reality trainer for ultrasound-guided regional anaesthesia. Anaesthesia 78(6):739–746. 10.1111/anae.16015 37010989 10.1111/anae.16015

[53] Arango S, Gorbaty B, Tomhave N, Shervheim D, Buyck D, Porter ST et al (2023) A high-resolution virtual reality-based simulator to enhance perioperative echocardiography training. J Cardiothorac Vasc Anesth 37(2):299–305. 10.1053/j.jvca.2022.09.004 36229288 10.1053/j.jvca.2022.09.004

[54] Rosenfeldt Nielsen M, Kristensen EQ, Jensen RO, Mollerup AM, Pfeiffer T, Graumann O (2021) Clinical ultrasound education for medical students: virtual reality versus e-Learning, a randomized controlled pilot trial. Ultrasound Q 37(3):292–296. 10.1097/RUQ.0000000000000558 34478430 10.1097/RUQ.0000000000000558

[55] Juliano JM, Schweighofer N, Liew S-L (2022) Increased cognitive load in immersive virtual reality during visuomotor adaptation is associated with decreased long-term retention and context transfer. J NeuroEng Rehabil. 10.1186/s12984-022-01084-6 36199101 10.1186/s12984-022-01084-6 PMC9532821

[56] Andersen SAW, Konge L, Sorensen MS (2018) The effect of distributed virtual reality simulation training on cognitive load during subsequent dissection training. Med Teach 40(7):684–689. 10.1080/0142159X.2018.1465182 29730952 10.1080/0142159X.2018.1465182

[57] Hall AK, Nousiainen MT, Campisi P, Dagnone JD, Frank JR, Kroeker KI et al (2020) Training disrupted: practical tips for supporting competency-based medical education during the COVID-19 pandemic. Med Teach 42(7):756–761. 10.1080/0142159x.2020.1766669 32450049 10.1080/0142159X.2020.1766669

[58] Ahmed H, Allaf M, Elghazaly H (2020) COVID-19 and medical education. Lancet Infect Dis 20(7):777–778. 10.1016/s1473-3099(20)30226-7 32213335 10.1016/S1473-3099(20)30226-7 PMC7270510

[59] Uschnig C, Recker F, Blaivas M, Dong Y, Dietrich CF (2022) Tele-ultrasound in the era of COVID-19: a practical guide. Ultrasound Med Biol 48(6):965–974. 10.1016/j.ultrasmedbio.2022.01.001 35317949 10.1016/j.ultrasmedbio.2022.01.001 PMC8743597

[60] Recker F, Höhne E, Damjanovic D, Schäfer VS (2022) Ultrasound in telemedicine: a brief overview. Appl Sci. 10.3390/app12030958

[61] Dreyfuss A, Martin DA, Farro A, Inga R, Enriquez S, Mantuani D et al (2020) A novel multimodal approach to point-of-care ultrasound education in low-resource settings. West J Emerg Med 21(4):1017–1021. 10.5811/westjem.2020.4.45928 32726277 10.5811/westjem.2020.4.45928 PMC7390551

[62] Blank V, Strobel D, Karlas T (2022) Digital training formats in ultrasound diagnostics for physicians: what options are available and how can they be successfully integrated into current DEGUM certified course concepts? Ultraschall Med 43(5):428–434. 10.1055/a-1900-8166 36198304 10.1055/a-1900-8166

[63] Fidler BD (2020) Use of a virtual patient simulation program to enhance the physical assessment and medical history taking skills of doctor of pharmacy students. Curr Pharm Teach Learn 12(7):810–816 32540042 10.1016/j.cptl.2020.02.008

[64] Kleinert R, Plum P, Heiermann N, Wahba R, Chang DH, Hölscher AH et al (2016) Embedding a virtual patient simulator in an interactive surgical lecture. J Surg Educ 73(3):433–441 26705061 10.1016/j.jsurg.2015.11.006

[65] O’Leary FM, Janson P (2010) Can e-learning improve medical students’ knowledge and competence in paediatric cardiopulmonary resuscitation? A prospective before and after study. Emerg Med Australas 22(4):324–329 20629697 10.1111/j.1742-6723.2010.01302.x

[66] Buch SV, Treschow FP, Svendsen JB, Worm BS (2014) Video- or text-based e-learning when teaching clinical procedures? A randomized controlled trial. Adv Med Educ Pract 5:257–262. 10.2147/amep.S62473 25152638 10.2147/AMEP.S62473 PMC4140394

[67] Thilakumara IP, Jayasinghe RM, Rasnayaka SK, Jayasinghe VP, Abeysundara S (2018) Effectiveness of procedural video versus live demonstrations in teaching laboratory techniques to dental students. J Dent Educ 82(8):898–904. 10.21815/JDE.018.086 30068780 10.21815/JDE.018.086

[68] Lebdai S, Mauget M, Cousseau P, Granry JC, Martin L (2021) Improving academic performance in medical students using immersive virtual patient simulation: a randomized controlled trial. J Surg Educ 78(2):478–484 32893155 10.1016/j.jsurg.2020.08.031

[69] Tan GM, Ti LK, Tan K, Lee T (2008) A comparison of screen-based simulation and conventional lectures for undergraduate teaching of crisis management. Anaesthesia and intensive care. 4 ed. United States. 565–9. 10.1177/0310057X0803600411 18714627

[70] de Lima Lopes J, Negrão Baptista RC, Takao Lopes C, Bertelli Rossi M, Swanson EA, Bottura Leite de Barros AL (2019) Efficacy of a video during bed bath simulation on improving the performance of psychomotor skills of nursing undergraduates: a randomized clinical trial. Int J Nurs Stud 99:103333 31450084 10.1016/j.ijnurstu.2019.04.001

[71] Starodub R, Abella BS, Hoyt-Brennan AM, Leary M, Mancini ME, Chittams J et al (2020) A comparative study of video lecture versus video lecture and high fidelity simulation for training nurses on the delivery of targeted temperature management after cardiac arrest. Int Emerg Nurs 49:100829 32029415 10.1016/j.ienj.2019.100829

[72] Mulcare M, Naik N, Greenwald P, Schullstrom K, Gogia K, Clark S et al (2020) Advanced communication and examination skills in telemedicine: a structured simulation-based course for medical students. J Teach Learn Resour. 16:11047. 10.15766/mep_2374-8265.11047 10.15766/mep_2374-8265.11047 PMC7751329 33365390

[73] Al-Balas M, Al-Balas HI, Jaber HM, Obeidat K (2020) Distance learning in clinical medical education amid COVID-19 pandemic in Jordan: current situation, challenges, and perspectives. BMC Med Educ 20(1):341. 10.1186/s12909-020-02257-4 33008392 10.1186/s12909-020-02257-4 PMC7530879

[74] Kasai H, Shikino K, Saito G, Tsukamoto T, Takahashi Y, Kuriyama A et al (2021) Alternative approaches for clinical clerkship during the COVID-19 pandemic: online simulated clinical practice for inpatients and outpatients-a mixed method. BMC Med Educ 21(1):149 33685442 10.1186/s12909-021-02586-y PMC7938264

[75] Daum N, Boten D, Schutz T, Sendeski M, Spethmann S (2021) Erwerb von Medienkompetenz zur Durchführung eines synchronen Online-Tutoriums zur Entwicklung fachlich-methodischer Basiskompetenzen in der medizinischen Aus- und Weiterbildung. Jahrestagung der Gesellschaft für Medizinische Ausbildung (GMA). Zürich, Schweiz. 10.3205/21gma142

[76] Hohne E, Recker F, Schmok E, Brossart P, Raupach T, Schafer VS (2023) Conception and feasibility of a digital tele-guided abdomen, thorax, and thyroid gland ultrasound course for medical students (TELUS study). Ultraschall Med 44(2):194–202. 10.1055/a-1528-1418 34225375 10.1055/a-1528-1418

[77] Barth G, Prosch H, Blaivas M, Gschmack AM, Hari R, Hoffmann B, Jenssen C, et al. Student Ultrasound Educaon, Current Views and Controversies; Who Should be Teaching? Z Gastroenterol 2024 (epub in advance). 10.1055/a-2356-7906 39074814

